# On the Rejuvenescence of Man, or Renewal of Human Life, and on the Means and Modes of Cultivating It; a Practical Treatise, Founded on Physiological Researches

**Published:** 1843-07

**Authors:** 


### ART. XIV.

*Ueber die Verjüngung des Menschlichen Lebens und die Mittel und Wege zu ihrer Kultur. Nach physiologischen Untersuchungen in praktischer Anwendung dargestelt von* DR. CARL HEINRICH SCHULTZ, &c.—*Berlin*, 1842. 8vo, pp. 445.

*On the Rejuvenescence of Man, or Renewal of Human Life, and on the Means and Modes of cultivating it; a practical Treatise, founded on Physiological Researches.* By DR. C. H. SCHULTZ, Professor of Medicine in ordinary at the University of Berlin.—*Berlin*, 1842.

THE idea of a constant renewal of human life, and of the possibility of maintaining perpetual youth, is as old as science itself. It has given origin alike to the study of personal hygiène and to numerous treatises on health and long life. It has occupied the minds of Aristotle and Bacon, of Paracelsus, Sanctorius, Hufeland, Kant, and others. Of the writers on the subject in our own country, Sir John Sinclair has perhaps obtained the highest celebrity.

Both the theory and practice of these authors have been faulty, even of the most celebrated and practical, as Bacon, Hufeland, and Sinclair. People must be treated according to their real and not according to ideal circumstances. Few men have the leisure, like Sanctorius, to weigh themselves for thirty years, or like Cornaro, the opportunity of taking their food and drink by weight and measure. Nor is it possible that the dietetic and hygiènic rules, so precisely and positively laid down by authors, can be acted on. Living in the world we must act with the world; and this same world is no rich valetudinarian ever fearing to transgress.

One of the fundamental principles of physiological science is that the materials of the bodies of animals are continually undergoing renewal; new matter being deposited and the old removed. The process by which the two acts are accomplished is termed by Professor Schultz the *verjüngungs* process, a word which may be translated as the *youthifying* process or the process of becoming young. If this process is completely interrupted, death takes place, and the more effectually it is performed, the stronger is the individual, and the more likely to have life prolonged. Hence the importance of a correct knowledge of its nature and its relations, and of the modes and means by which it may be maintained; or if interrupted, may be restored. This is the object of Professor Schultz's inquiries.

In the present article we purpose giving a pretty full account of the author's views, restricting ourselves, however, almost entirely to an analytical summary of them. If we did not think some of the doctrines very important, and yet more of them ingenious, we would not trouble ourselves or our readers with any detailed exposition of them; but it would occupy much more of our space than we can at present afford to discuss them critically, or to point out the numerous instances where we demur and where we differ. On another occasion we may resume the subject; at present we hope we may claim not merely the attention of our readers to our analysis, but their thanks for the trouble we have taken to gratify them. It is only the *labor ipse voluptas* of a British and Foreign reviewer, that could minister to them in this kind.

The general process comprehended in Professor Schultz's inquiries, is subdivided into two others : *a*, the process of reorganization ; *b*, the process of disorganization, and separation of the effete molecules from the body, or *moulting* (Mauser), as our author prefers to express himself. These two are the poles between which the pendulum of vital action swings, the poles of life and death, of renewal and destruction, of revival and effeteness. And as the whole term of life is periodic, so is the alternation of these processes. In short, in the renewing process *we have to trace a current of vital changes through the channel of periodicity.* Illustrations of the processes are drawn from the moulting and other phenomena of insect life, and from the development of plants. The moultings of insects are not confined to the epidermis, but extend to all the mucous membranes; the matter thrown off at each change being the *debris* or effete matter remaining after the renewal of the tissues. These changes in moultings are traced to the point at which the generative organs become fully developed, and ova are deposited; when the *verjüngungs* or renewing process is exhibited in the production of a new animal, and the parent is itself the debris or educt of the final moult. (!) A perennial plant so called is really not a perennial at all, but merely a whole generation of twigs and layers of wood and bark springing annually from the old stem. The inner structure of the tree never changes. No plant can be more than biennial, as this is the utmost period required by any for the development of the blossom and the ripening of the fruit. What remains after the latter process is accomplished is merely the residue of a life or lives ended. An old tree is like a coral reef, a monument of death formed out of the debris of the successive generations which have annually sprung from it. And a strict analogy may be traced between the blooming of plants and their death after fructification, and the development of the generative organs of insects during their successive metamorphoses and their death after the deposition of their ova. In both the development of the individual arrives at the same results and exhibits the same effects. In animals of a higher grade the complete development of the generative organs, and the renewal of the species do not occur contemporaneously with a total cessation of the renewing process in the parent; but the general law is manifest in the moultings of the cutaneous appendages to which they are liable, and which occur contemporaneously with changes in the ovaria and testes. The substances moulted are but the debris left by the renewing process, and the changes in both the skin and generative organs are only indicative of the moult and the renewal occurring throughout every part of the system of the individual. The mucous membrane of the lungs and alimentary canal, and of the genito-urinary apparatus, moults as well as the skin. The teeth in many fishes and reptiles are a product of and fixed into the mucous membrane, and are thrown off when the general renewing process takes place, just as are the cutaneous appendages. Indeed the teeth generally have an important relation to the periodic renewals and changes in the individual.

Such are our author's views as regards the renewing process in general; and now we come to more special changes, namely, to the death and renewal of the microscopic structures, and especially of microscopic constituents of the blood. Our author's inquiries as respects the latter are very curious, very novel, and, if they prove to be correct, will be of the highest value in practical medicine.

Professor Schultz observes that the attention of physiologists has been concentrated rather upon the chemical and mechanical composition of the blood than on its organic properties. A knowledge of its vital phenomena cannot, however, be attained either by dynamics or chemistry, but only by an organic analysis. This analysis Professor Schultz has already undertaken and given the results to the public.* In the present work these results are farther developed and carried out to practice.

"We have shown that the organic constituent of the blood, namely, the blood-vesicles, the so-called blood-corpuscles, are by no means unchangeable and fixed constituents as they have hitherto been considered; but are being continually re-formed and decomposed; that both in the embryo and during digestion they continually originate anew and displace the old; and that when arrived at the climax of development they begin to dissolve and die, their debris being invariably separated from the body as the new vesicles form. In the blood of an animal, or man, blood-vesicles may be found in all stages of development between their first origin and dissolution. I believe I am the first who has given a history of the development of the blood, and shown in what the vital activity of the blood exists. These researches have rendered us better acquainted both with the true organization of the so-called blood-corpuscles as well as of the plastic constituent of the blood. We can also better understand the part taken by these constituents as well in the formation of the blood itself as in the formation of the body. We can distinguish between their vital manifestations and their chemical properties. We can penetrate into the connexion between their vital activity and other functions; and, lastly, we can study the pathological changes which are dependent on, or co-existent with, abnormal vital action in these organized constituents." (p. 37.)

*Composition of the blood.* According to our author's researches there are only two organized constituents in the blood; namely, the plasma and the vesicles. Neither fibrine nor serum exists as such in living blood. The latter is formed as a chemical product during coagulation; the former is a product of the vital power during the death of the blood. The plasma corresponds to the liquor sanguinis; it may be separated from the blood by two or three methods. The best is the following: let the blood flow directly into a tall glass cylinder, and when full close it quickly so as perfectly to exclude the air. By this means the plasma is kept longer fluid, and time is given for the vesicles to sink to the bottom of the cylinder, leaving the plasma supernatant. Or the blood may be collected, by means of a funnel, into a piece of intestine previously well cleaned, and closed at one end. If the piece of intestine be made perfectly full and the open end closed tight with strong twine and then hung up, the vesicles will separate from the plasma, sinking to the most dependent part, and the intestine can be bound with thread at the point of separation between the two.

*Composition and properties of the blood-vesicles.* The vesicles of vertebrate animals in their perfect condition consist of an outer mem-

* 1. System der Circulation in seiner Entwickelung durch die Thierreiche und im Menschen.—Stuttgard, 1836. With Plates.

2. Der Lebensprocess der Pfortader systeme. Hufeland's Journal der pract. Heilk. Feb. 1837.

3. Ueber die gehemmte und die gesteigerte Auflösung der verbrauchten Blutbläschen. —Ibid. March, 1838.

brane, containing an elastic fluid in which a granule floats. This membranous cell is at first colourless, but afterwards becomes coloured by the colouring matter entering into its tissue and rendering it opaque. If fresh vesicles be placed in brine or a weak saline solution and water be added gradually, the colouring matter will be dissolved out of the vesicles and they then become thin, transparent, and almost invisible. But their texture is not altered, for Professor Schultz has found that by adding tincture of iodine to the solution the vesicles become dark-brown. (p. 40.)

In making experiments on these vesicles various interesting phenomena may be observed. 1st. The same quantity of water has a different effect upon different vesicles. Some become quite colourless, others only imperfect, others are unchanged. This difference depends upon the quantity of colour contained originally in the membrane of the vesicle; those having a larger quantity requiring a larger quantity of water to abstract it all. The quantity of colouring matter in the vesicles varies in different classes of animals. In the vesicles of the blood of fishes it is the least; in birds and mammalia the greatest. In the vesicles of all embryos there is less than in adult animals. In all the quantity is in relation to the stage of development of the vesicle. 2d. The perfectly developed vesicles of the vertebrata and of man are flat so long as they contain their colouring matter. When this is removed by water they then assume a globular form, and the inner part of the vesicle and its contained granule become more distinct. 3. The vesicles are remarkably elastic, particularly in their colourless state. 4. They possess an organic contractility during life, easily demonstrable by certain stimulants, as cold, neutral salts, and alcohol. This contractility is very obvious when vesicles deprived of colouring matter are brought into contact with salt; they then become flat again, and are also contracted into a variety of forms. In the perfect vesicles it is less obvious. When the vesicles die they are no longer contractile, and become quite flaccid. In this state they lose the capacity of dilatation, so that it cannot be by the dynamic property of endosmose that the living vesicle is filled. This, according to our author, is the basis of the vital power of the blood. The excitability imparted during respiration is founded upon it. It is extraordinarily great in the young and newly-formed vesicles found in the embryo and the lymphatics. The more active the contraction of the membrane the less readily is the colouring matter withdrawn from it, and this explains why it has been generally supposed that the colouring matter is not soluble in saline solutions of a certain strength, the salt acting as a stimulus to contractility, and consequently rendering the separation of the colouring matter less easy. In living blood the vesicles are in a state of natural contraction from the oxygen contained in them, and from the salts held in solution in the plasma.

With reference to the statement that the vesicles contain oxygen gas, Professor Schultz observes that there has hitherto been considerable uncertainty as to the presence of gases in the blood. He demonstrates and collects it thus. He takes as large a bottle as he can get, provides it with an air-tight stopper, and fills it full of blood fresh drawn from a horse until it runs over. The stopper is then thrust in, and the blood set aside to cool. As this process goes on a vacuum is formed at the upper part of the bottle, and little globules of gas are seen collecting from all parts of the mass into the vacuum. The chemical analysis of this gas shows that it is oxygen gas if arterial blood has been used for the experiment; carbonic-acid gas if venous blood. Professor Schultz infers from these researches that the oxygen gas in the lungs is absorbed into their interior by the vesicles. The capacity to absorb the gas depends upon the degree of excitability in the vesicles; those with large granules have the greatest separating power. In general the smaller the granule and the more the membrane is coloured the less the contractility, and *vice versâ;* large granules with almost colourless membranes have the greatest contractility. These are found in the greatest number in the rose-coloured lymph in the thoracic duct.

*Origin, development, and death of the blood-vesicles.* The first origin of the vesicles is seen in the lymphatics. The lymph contains naked granules of different sizes, the so-called lymph-globules. The largest of these are perfectly soluble in ether; it is obvious also, from their smooth shining appearance, that they are fat-globules. They also reappear when the ether is evaporated. The larger lymph-globules become metamorphosed into the smaller, and round these a filamentous vesicle is seen to be developed, which is at first perfectly globular, colourless, and transparent. In others in a more advanced stage it is seen that the membrane begins to be coloured, and in these contractility is developed. The lymph-globule is closely shut up within the membrane, so that the blood vesicles are in reality formed in the lymph, and their granules are fully-developed lymph-globules. When the vesicles thus formed pass into the current of the circulation they are quickly subject to further changes. The more frequently they pass through the lungs and are subject to the influence of oxygen gas, the more the size of the granules diminishes, until at last they disappear altogether from the vesicle. And in proportion as this change takes place the vesicle becomes more deeply coloured and less contractile until they are quite dark and without contractility, becoming *pari passu* specifically heavier than the plasma. By means of the latter property the vesicles of different sizes may be separated from each other, as when blood is taken into a glass cylinder, the oldest being the most deeply coloured and the heaviest sink to the bottom, and the youngest and least coloured are at the top.

When the blood-vesicle has become incapable of being further acted on by oxygen gas, it is useless. The colouring matter must be removed, and the residuum, or film be excreted or reorganized. According to Professor Schultz it is in the liver these changes take place. The absence of valves in the vena portæ, and the slow motion of the blood, are favorable to the precipitation of the old heavy useless vesicles from the general current of the circulation, and the more fluid plasma readily extracts the colouring matter of the flaccid films. On a chemical analysis of the portal blood, the plasma is found to be less in quantity and more fluid, and containing more colouring matter than that of venous blood. This must necessarily be the fact, because the vesicles part with their colouring matter the more readily as they become less contractile. So that in the vena portæ two things take place: 1. The old useless vesicles are taken out of the circulation. 2. The debris, or dead films of these vesicles are separated from the blood.

*Function of the blood-vesicles.* According to Professor Schultz's researches, the blood-vesicles have no direct connexion with the nutrition of the body, but are the true respiratory organs of the blood, and subservient to the completion of the process of assimilation. By the absorption of vital air, the granular substance is transformed into plasma, and the colouring matter is the residue of the transforming processes. It is by no means necessary that the respiration of the blood should be performed in lungs or gills: any surface may suffice to bring the vesicles in contact with the atmosphere, and in some animals the whole surface of the body is a respiring organ.

*Nature and origin of the plasma.* The plasma is the colourless organized plastic fluid in which the vesicles float. It is the coagulating portion of the blood. Coagulation takes place more quickly and perfectly in proportion as the vesicles are deposited. This process is not chemical, as is the coagulation of albumen, but a vital act developed in the death of the blood. The plasma is formed during the development of the granules of the vesicles with the aid of respiration. It is the true formative and nutritive material in the blood, and supplies all the structures, passing through the parietes of the vessels into the parenchyma of organs, which the vesicles never do. Consequently, there is less plasma in the returning venous blood than in arterial. The plasma takes up matters passing into the circulation. Indigo colours it green; but the blood-vesicles remain unchanged. The vesicles carrying oxygen excite the muscular and nervous system, the plasma is directed rather to the vegetative system.

The following are some of the pathological inferences Prof. Schultz deduces from these researches.

If the old deeply-coloured vesicles are not excreted from the circulation as new ones form, the blood assumes a darker tint, and the portal system is congested, because the old vesicles accumulate in the liver. And as such vesicles cannot undergo the necessary changes in the lungs, and carry oxygen into the system, a necessity for increased respiration is excited, and asthma and dyspnea are developed. The natural excretion of the vesicles may be altered by a want of tone and contractility in the containing membrane of the vesicle, as in chlorosis; or in a more saline or aqueous state of the blood, as in dropsies: in both diseases the formation of plasma is interrupted. The plasma may be also diminished in quantity from an imperfect development of the granules, itself dependent on imperfect digestion and assimilation; as in scrofula and scorbutus. In the lymphatic temperament the blood-vesicles develop themselves slowly, and the blood contains a large proportion of old vesicles. In the sanguineous temperament they run their course more quickly. Naturally the plasma is colourless and transparent, but in man and horses suffering from phthisis it is opaque and quite milky. Vegetable substances, as indigo, act upon the plasma: iodine has a special action on the membrane of the vesicles, staining it permanently of a brown colour, hardening it, and destroying its contractility. Neutral salts act on both the vesicles and plasma: narcotics on the vesicles only, destroying their contractility.

Let us now revert to the general views of our author. The last change the blood-vesicles undergoes is a *moulting.* Each vesicle has a cycle of life precisely as each individual animal; and every vesicle has equally its birth, course of development, and death. The dead debris of each also must, like the moulted appendages of the skin in insects and vertebrata, be thrown off. The blood purifies itself from them, and the liver is the organ in which their exit from the circulation is made.

This process of depuration is not a purifying of the blood from foreign matters, but a self-purification from the moulted refuse thrown off during its own development. It is a true moulting of the blood itself, and as necessarily connected with the renewal and rejuvenescence of the blood, as is the cutaneous moulting of animals with contemporaneous renewal of other organs. It is a renewal in virtue of the periodicity of the vital processes carried on in the blood, and altogether independent of the renewal of unassimilated matters derived from without. It is not gaseous matters that are here excreted, but the fixed or solid residues of the coloured membranes of the vesicles which have passed through their cycles of existence. As the apple falls from the tree when it is ripe, as the leaf is loosened when dead, as the uterus expels the fœtus when at its full development, so the blood is moulted from those old vesicles that have fully completed their development. Since the vascular system has no direct emunctories through which the moulted debris may be thrown off, the circulation through the liver pours them out into the intestines as bile. The term vena portæ is prophetic, as that vein is a porta, a gate, in more than one sense.

We cannot follow our author through his researches into the functions of the liver, and the composition of the bile, made with reference to his peculiar views. His proof of the correctness of the latter are drawn from comparative anatomy and physiology and from direct experiments. The composition of the blood in the vena portæ is proved to differ from common venous blood; its colouring particles are darker, and are less affected by oxygen or neutral salts; it contains less plasma, and its plasma contains colouring matter; it has less albumen, and $1\frac{1}{2}$ and $2\frac{1}{2}$ per cent. respectively of fibrine more than venous and arterial blood. It also contains a greasy dark-brown fat, not found in either. It is nevertheless more aqueous, and its motion through the veins is slower, evidently from the diminished quantity of the plasma, since by the plasma the motion of the blood in the vessels is maintained. Professor Schultz observes that the colouring matter obtained from venous, arterial, and portal blood respectively, has peculiar qualities, and that the colouring matter of the portal blood is closely allied in chemical composition to the bile. Portal blood, however, is not wholly composed of moulted venous blood, as the lymphatics take up a residue from the chyle.

*Renewal of the muscular and nervous structures.* We readily agreed with Professor Schultz in his assumption that the muscles and nerves are renewed, and looked anxiously for some account of the microscopic changes they undergo during the process of renewal. In this we have been disappointed. As the muscles and nerves moult like all other organs, or in other words cast off the animal matter rendered effete by subserving to functional activity, there must be an organ or organs by which the debris are thrown off. These, according to Professor Schultz, are the kidneys and the perspiratory organs of the skin; and various facts are brought forward to substantiate this opinion. There are the close relations between rheumatism and other muscular affections and the skin; and between the nervous system and the urinary secretion. The changes in the latter are very obvious in many affections: as for example, in all spasmodic affections, in diseases of the spinal cord, and in many hysterical disorders. It is not, however, every constituent of the urine which can be considered as the moulted matter of the nervous system, but exclusively urea and uric acid, and their modifications and compounds. These only are affected by increased or diminished activity in the nervous system, and the process of neurine renewal. The great similarity between the chemical composition of these substances and of the albuminoid matter of the nerves renders it also probable that the former are simply the result of a metamorphosis of the latter; just as the purpuric and cyanuric acids, and the urate of ammonia are only metamorphoses of urea and of uric acid.

The symptoms following the retention of urine in the system are such as might be expected on the supposition that it is the moulted matter of the nervous system. The critical urine of intermittent fever and of gout is explained as an illustration; as also the great diminution in the secretion of urea during the presence of spasmodic diseases. Nysten showed experimentally that in these cases there is only 1 per cent. instead of 3½ per cent. of urea in the urine. Professor Schultz refers to Dr. Addison's researches on the connexion between cerebral affections and those diseases of the kidney first clearly exhibited by Dr. Bright. He might with propriety have referred to the published opinions of Dr. Holland, Dr. Copland, and Dr. Laycock: to the two former for arguments as to the connexion between gout and certain constituents of the urinary secretion, and to the last mentioned for researches into the connexion between lesions of the urinary system analogous to the gout, and many anomalous nervous affections of females. (See Br. and For. Med. Rev. vol. XII. p. 73.) Pathological facts of this kind are numerous, and were well known to the old humoral pathologists. What the modern humoral pathology wants is a microscopic analysis of the vital changes in the blood, and those occurring during muscular and nervous action, and in the renewal of the muscle and nerve.

*Renewal in relation with the respiratory process.* Professor Schultz refers to his published Elements of Physiology for a demonstration of his opinion that the final cause of respiration is to revive the blood-vesicles after their circulation through the system and to exalt their development. It is not the lungs, but the blood which respires by means of the lungs; and it is the blood-vesicles which inspire and expire as the blood-lungs. The air taken into the lungs acts upon the granules of the vesicles, forming them into plasma, the true nutritious and life-exciting constituent of the blood. The life of the blood is at its climax during this metamorphosis. It is by a vital tonic power that the blood-vesicles draw in the air. This power commences in the lymphatic system, when the membrane or film first forms round the lymph-globule, and by which it is enabled to develop itself further, and act upon external agents just as the ovum when vivified is enabled to act on external agents. The cycle of life of the blood-vessels is from the alimentary canal through the lymphatics, and the venous system to the lungs; from the lungs through the arterial system to the portal system; and from thence through the liver to the alimentary canal, where the process of vitalization recommences. Thus the blood is ever renewing, ever revitalizing itself.

*Pathology of crises.* Health consists in a balance between the two processes of reorganization and disorganization; disease in a preponderance of the latter over the former. The moulting of diseases is the return to due vital action ; the renewal of health by the regeneration of the effete parts in the suffering organ. When this regeneration is normal, there are crises in the evolutions of structures : of this kind are the moulting of birds, the renewal of the hair in mammalia, the casting of the skin and scales in reptiles, the metamorphoses of embryos and insects. A crisis considered as an excreting process is only the termination of the moulting process. This analogy between the stages of disease and the natural processes going on in the system is carried out into details and the scholastic theories of crises, as coction and the like, are explained.

*Relations of healthy and morbid renewal.* Just as the natural processes lead to diseases so the morbid moultings or renewals lead to health. The natural exuviation and renewal may be interrupted in various ways. Thus, the interruption of the blood-moulting in the portal system may induce abdominal infarctions ; interruption of the moulting of the skin may bring on pulmonary or hypochondriacal affections; of the lungs, may induce atrophy; of the alimentary canal, diseases of the brain and nervous system. Therapeutics consists in an artificial excitement of the natural exuviation. For example, artificial excitement of the exuviation and excretion of the blood-vesicles in the portal system may regenerate the whole digestive and nutritive processes. Diseases themselves often induce this favorable exaltation of the process. The hair, nails, and epidermis are thrown off, the epithelium of the alimentary canal removed, the effete debris of the blood-vesicles thoroughly expurgated from the portal system through the liver, and so the whole machine becomes in some degree new formed. Thus nature, in spite of science, renews the body after her own fashion.

Some phenomena resulting from interruption of the natural processes may be mentioned in illustration of our author's views. 1. The vital renewal may be imperfect, and the new formation, just as the embryo, may be an abortion, or may be imperfectly developed. This occurs in scrofula, in which the constituents of the lymph are arrested in the first stage of development, giving rise to irritation and an inflamed condition of the lymphatic glands. In chlorosis, the blood-vesicles are arrested in the lymph-stage, and remain pale, their membranes being as irritable as in fishes. The formation of the plasma is consequently imperfect, and there is a want of energy in the whole of the formative process. In persons of a phlegmatic temperament the muscles are arrested in the cellular stage of organization : in children of the nervous temperament the substance of the brain and nerves is not fully developed, the albuminous basis being imperfectly assimilated. 2. The vital action may be excessive, and a high degree of plasticity result, as in tonic plethora.

*Cultivation of the renewing process.* This is the heading of the third division of Professor Schultz's volume. The general principles which should guide us are first laid down. As might be inferred *a priori*, it is a fundamental rule that the equilibrium between reparation and destruction be maintained. Another general rule is that this equilibrium be maintained consonantly with the natural periodicity inherent in the system. The cause of this periodicity of vital change is exoteric.

"Excitement and repose, exhaustion and restoration, increase and diminution of functional activity, assimilation and resorption, all alternated in periodic change, determine the general laws of vitality. It is not an undefined general principle that is in operation, but an actually operating antithesis of vital functions with which we have to do. The periods are to be determined less by telluric periods, as the times of the day, than by the periodic activity occurring in the organism itself." (p. 111.)

With respect to the periods so often referred to by our author we may observe, that henceforth the first duty of an inquirer into the subject must be to determine the length of the periods. The fundamental idea of number once fairly introduced into physiology will be like a mental telescope or microscope to the philosophical inquirer. It will enlarge his sphere of intellectual vision, and give precision of outline to the general facts he may contemplate. The determination of the exact periods of moulting in insects, or of incubation in oviparous vertebrates, considered as stages of development, would be worth many volumes of vague statements and ingenious speculations.

*Cultivation of the process of assimilation.* We pass with a feeling of satisfaction from the preceding speculations to the practical rules respecting diet and regimen, and the proper culture of the digestive organs laid down by our author, and shall notice a few of the most remarkable of his observations and experiments.

1. The stomach should be well filled when eating, so as to press against the diaphragm, and abdominal muscles. This rule is inferred from experiments instituted by Professor Schultz leading to the conclusion that the rotatory motion of the stomach is essential to good digestion, and that this motion is most energetic when the viscus is well supported by the diaphragm and abdominal parietes.

2. The stomach should be perfectly empty before food is again introduced, because according to the laws of periodicity, a period of repose should always follow a period of activity. The reaction of hunger may be taken as a general indication of the necessity of a further supply of food: not always, however, for it is sometimes morbid, just as the perception of sounds, colours, &c. If the stomach be well filled, the digestive process requires from six to eight hours for its completion.

3. Suppers are not to be recommended, particularly in diseases of the large intestines. Professor Schultz adheres to his old original idea that in adults the cæcum performs the function of a second stomach, digesting the alimentary matters which from their hardness or other circumstances are not sufficiently acted on in that viscus. This cecal digestion is in every respect analogous to the ventricular process, requiring like it the proper digesting fluid, and particularly the bile, for its full completion. It is carried on principally in the evening; and since the liver cannot serve two masters, if the stomach be filled at night, the digestive process in the large intestine is interrupted.

4. The sensation of faintness in those subject to indigestion is increased by frequent eating, and cured most readily by fasting. This sensation, according to Professor Schultz is dependent upon an imperfect chylification, and consequent on this is imperfect sanguification, and this imperfect chylification is induced by interrupting the ventricular and cecal digestion before the completion of the process, which too frequent ingestion of food will do.

5. Food should be properly cooked, for gross feeding and gross minds go together.

6. Professor Schultz contends, contrary to general opinion, that boiled meats are more digestible and nutritious than baked or roasted, and refers to his work *De Alimentorum Concoctione* for experiments in proof. For similar reasons he asserts that the invertebrate animals and cold-blooded fishes and reptiles are less digestible than warm-blooded animals. Fowls are more digestible than beef or mutton, and so indeed is pork. But much depends upon the mode of cooking and the age of the animal. Fat is very digestible, and not so liable to become rancid as is generally thought; it is also exceedingly nutritious. A case of consumption, according to Professor Schultz, was cured by the methodical use of goose-fat as an article of diet, although a great part of the lungs was destroyed. Cheese ranks after flesh in point of digestion: it has also the property of promoting the assimilation of other substances. Our author recommends it to be taken with oysters. Milk and eggs (which contain the milk of birds without the sugar) are not commendable as articles of diet.

We must pass over some judicious remarks on water and manufactured drinks, to make room for Professor Schultz's experiments respecting the physiological and pathological action of alcohol.

*Action of alcohol on the blood.* "Since physiological researches have shown that alcohol taken into the stomach is in great measure absorbed from the alimentary canal, and carried into the circulation unchanged, physicians have directed their attention exclusively to its action on the mass of the blood. The special changes excited by alcohol on the individual constituents of the blood have not yet been ascertained. It has been observed generally that the blood becomes of a more venous character, and contains more carbon and hydrogen, not however specifying which part of the blood suffered the change, whether the blood-vesicles or the plasma...... The chemical changes have certainly been inquired into, but without a knowledge of the fact that it is in the organic constituents and microscopic elements of the blood the true nature of the changes is to be found...... If a little spirit of wine be added to fresh blood a dark tint becomes perceptible to the naked eye. But upon closer examination it will be found that it has not really become darker.... If it be examined with a microscope it is found that the colouring matter has changed its locality: that it gradually passes from the vesicles into the plasma, and that the former appear at last as perfectly transparent, colourless bodies swimming about in the fluid of the plasma, or, if coagulated blood have been used, in the serum. The relations of the two constituents of the blood are in fact reversed, and instead of coloured vesicles swimming about in the colourless plasma, we have a uniformly red-coloured plasma and colourless vesicles. The colouring matter is chemically held in solution in the plasma, and consequently the darker shade of that fluid is only *mechanical* (?) When spirit of wine is added to fresh coagulable blood the red plasma changes into a gelatinous matter of the consistence of thick milk, no formation of clot or serum subsequently taking place." (p. 171.)

Professor Schultz contends that the analogy between the action of diluted acids and alcohol on the blood is more apparent than real. The acids coagulate the albumen, but the colouring matter is not acted on by the alcohol, unless it be mixed with the blood in large proportions when the albumen is coagulated. The decolorization of the vesicles by alcohol does not take place immediately but gradually, and is more or less perfect according to the quantity used. If the contraction in the vesicles excited by the alcohol be kept up continuously, the vesicles contract to the size of a point, and at last disappear altogether, and the blood exhibits the appearance of a uniform transparent red fluid, without perceptible red globules. The newly-formed young vesicles contract the most energetically; the older and more deeply coloured resist its action longer. The vesicles that have become perfectly colourless and quite contracted exhibit only chemical properties, and have lost all vitality and excitability. The action of narcotics on the blood is very different from that of alcohol. Narcotics destroy the contractility of the vesicles so that they are paralysed, and remain expanded, and the accumulation of the colouring matter takes place in the vesicles only.

The action of alcohol then on the blood is not chemical but vital. It acts as a stimulant to an increased and unnatural contraction of the vesicles by which they are deprived of colouring matter, and hurried on to the last stage of development or death. Now, since the activity of the respiratory process depends upon the vital activity of the vesicles, it is a necessary inference that less oxygen will be absorbed and less carbonic acid gas excreted in proportion as the vesicles are devitalized; and this is the true explanation of the venous character peculiar to the blood of drunkards. The formation of plasma, the true nutritive material of the blood, is interrupted *pari passu* as the respiratory process is interrupted, and the plasma itself becomes an irritant to the circulatory and secreting organs from the presence of the colouring matter of the vesicles. So that while on the one hand congestion is taking place in the capillaries, there is an unnatural irritation of the secreting organs on the other; the necessary result being interruption of function.

*Action of alcohol on the spleen and liver.* The explanation of the enlargement of the spleen observed in drunkards is connected with a theory of our author's respecting the functions of the spleen, in virtue of which that organ is considered a part of the lymphatic system, and subservient to the depuration of the blood. The digestive process being interrupted by the change in the organic constituents of the blood, the food is imperfectly assimilated, and raw nutritive matter carried with the chyle into the circulation. The office of the lymphatic system in the spleen being to further assimilate this raw material, that organ becomes overfilled with blood in proportion as the unassimilated constituents of the chyle increase.

Professor Schultz observes with respect to the liver that there is a double series of phenomena. First, the lymphatic system of the liver is placed in the same abnormal position as that of the spleen: and secondly, there is portal congestion. The change in the biliary system is connected with the general changes in the blood already referred to. It is not simply the effete carbonaceous colouring matter which is transferred from the vesicles to the plasma, but the arterial oxygenizable colouring matter also, and the latter is as unfit for the formation of bile as the blood of the hepatic artery itself.

Professor Schultz connects the pathology of delirium tremens or drunkard's madness, with the physiological changes excited by alcohol in the blood. Unnaturally stimulant plasma on the one hand, and the disorganized vesicles on the other lead to a state of asthenic irritation, a subinflammatory and congestive condition of the brain and spinal cord. It is not an exhaustion of these organs from excessive stimulation that is the cause of the delirium: it is rather a qualitative disorganization and destruction of the exciting process induced by the change in the exciting powers of the blood.

Professor Schultz questions very much the correctness of the principles of total abstinence so enthusiastically advocated by the "tee-totallers." The practice of taking stimulants is so generally diffused, and it is of so great antiquity that it may be considered instinctive. If men eschew brandy, they will chew opium ; or if they eschew opium, they will chew betel, or tipple with wine. The tee-totallers, Professor Schultz thinks, may possibly do more harm than good, or to use his own expressive phrase, may lead us out of the rain to the spout, and German spouts *are* water-spouts in a raining day. He thinks it a better plan to render spirituous drinks less injurious by assimilating them to wine; in fact, by encouraging the manufacture of made wines, for our author argues that wine is comparatively innocuous from its peculiar composition. He gives the following recipes for two kinds of made wines; we should call them punch.

*Citron wine.* The juice of twelve citrons: two quarts of spirit, and four quarts water: one pound of sugar. Mix together, and set aside for several weeks until clarified and ether is formed.

*Essig-ether wine.* Four ounces of wine-vinegar: two quarts of spirit at 80: four quarts of water: two pounds of honey, or one pound of sugar. Set aside until ether is formed.

*Culture of the digestive process.* Professor Schultz thinks that late are to be preferred to early dinners, and commends the English and French habits as being superior to the German, and closely resembling the ancient Roman custom in this respect. An early dinner involves the necessity of a supper, and supper (as has been already shown) interferes injuriously with the cecal digestion, especially in adults. The Romans had five legitimate meals: breakfast (jentaculum), lunch (prandium), tea (merenda), and supper (cœna); the fifth being a jollification after it (commissatio). But only one of the five was a substantial, physiological meal. The *jentaculum* was taken by children alone. The *prandium* was only a snack, and as for the *merenda* and *commissatio*, the one was exclusively taken by slaves and labourers, and the other by the drunken sots who in modern phrase are termed jolly good fellows. But was the Roman cœna a five-o'clock, or a six-o'clock, or an eight-o'clock dinner? and if an eight o'clock dinner, how can Professor Schultz lay blame to his countrymen for dining at half-past one, and supping at half-past eight? Probably, a dinner at five or six o'clock is preferable to any other; at a later hour, it would be a supper, at least physiologically ; at an earlier, a *bona fide* supper would be necessary. A six-o'clock dinner gives the stomach time to be prepared for a substantial jentaculum at nine next morning:—a true Roman prandium, an Abernethy biscuit and a glass of wine and water at one will keep it from grumbling until six. The choice between a commissatio and converzatione is of course a matter of good taste and morals, except to the invalid.

*New experiments on the physiology of digestion.* Considering how much health and long life are dependent upon a due culture of the assimilating powers, Professor Schultz instituted a series of experiments on the physiology of digestion. He observes very justly, that the generally received opinions respecting the agency of the gastric juice are very unsatisfactory. They give no good account of mastication or rumination, or of the part the saliva takes in digestion, although digestion is performed in the mouth in many amphibia, and other animals have salivary glands in the stomach itself. Before relating the new experiments of our author, we shall give the results of those already published in his work *De Alimentorum concoctione :*

"1. That, excepting during digestion the secretions of the stomach are always alkaline; they are even alkaline at the commencement of digestion in many animals, and in those which fast long, as the hybernants, the contents of the intestinal canal are alkaline also.

"2. That the degree of acidity in the chyme in the different divisions of the stomach corresponds closely with the stage of digestion, is altogether different as the food is different, and becomes less as digestion is prolonged.

"3. That there is much less acid formed in the stomach during digestion, than is requisite to chemical action on the food. Chyme from vegetable food requires from one to one and a half per cent. of carbonate of soda for saturation; chyme from cheese requires from 1 to 1¾ per cent.; from flesh 2 to 2½ per cent. All the chyme in the stomach of a calf, amounting to about four ounces, was saturated by less than half a drachm of the carbonate. From forty to sixty grains at the most will saturate any quantity that may be found in the stomach of a dog.

"4. That the stomach like all other mucous membranes secretes mucus only, but is specially adapted for absorption of fluids: consequently, the stomachs of fasting men and animals are quite empty.

"5. That there is a great quantity of saliva being constantly secreted and also swallowed, even when digestion is not going on; that in animals furnished with a stomach having a horny lining, the saliva thus swallowed collects in considerable quantities: that in animals in which the mucous membrane of the stomach is naked, the saliva becomes concentrated by the absorption of the watery portion, leaving the fixed constituents in the stomach. Many ounces of this concentrated saliva may be found in the empty stomach of the horse. The parotid gland of a single horse secreted more that 100 ounces of saliva in twenty-four hours.

"6. That, consequently, all fluids found in the stomach, or extracted from the mucous membrane necessarily contain salivary matter, or rather saliva in a concentrated state; and also, that all gastric juices which have been collected or prepared for experiments contain the salivary constituents.

"To these statements may be added—*a.* That, as Spallangani well knew, no food can be digested artificially without saliva. *b.* That the chyme is not a chemical solution but an organic compound formed by the transformation of the constituents of the food; as for example, starch being transformed into sugar, sugar into acids: changes which never take place in the common experiments on artificial digestion. *c.* That the acid of the stomach is a product of digestion: that the alkaline contents of the stomach are first saturated before free acid is present, and that the latter during fasting gradually disappears, and an alkaline condition is restored; the period required for the completion of the changes varying in length from six to twelve hours. *d.* Permanent acidity is only observed in diseased states of the stomach, as in heart-burn, in which the digestion is impaired, although according to the chemical theory it ought to be energetic. Fermentation and the formation of carbonic acid gas, which never take place in healthy digestion, result from this diseased condition. *e.* The ingestion of acids during digestion interrupts that process under all circumstances.

"From all these statements it follows that the fluid which physiologists have termed the gastric juice, is only a medley of various things taken into the stomach, and not, with the exception of the mucus, secreted by it. The acid is a chemical product from the food produced as the latter is continually agitated with the chyme. But above all, the acid gastric juice obtained by experimentalists from healthy men and dogs contained acid chyme. *There is, in fact, no such thing as acid gastric juice, but only sour chyme.*" (pp. 187-190.)

The flat contradiction here given generally to the inferences from Dr. Beaumont's experiments on the Canadian voyageur is followed up in a note by a special reference to those experiments. This contradiction is, however, pushed to its utmost limit when our author declares, in the very teeth of Dr. Beaumont's conclusions, that the saliva is the main agent in the digestive process. As this view has been advocated from experiments by at least one physician in England, we refer to Dr. Wright, and as it is also founded by Professor Schultz on carefully-made experiments, we shall notice it in detail as far as our crowded volume will allow.

*New experiments on the digesting power of the saliva.* Prof. Schultz first prepared the saliva used in these experiments by concentrating it, for some, to the consistence of thin syrup; for others, to a state of perfect dryness. In the latter state it was reduced to a white friable mass, containing nevertheless the true digesting element; and formed when rubbed up with five or six parts of water a milky fluid, having the same proportion of fixed constituents as the less concentrated saliva, but constituting a fluid less effectual than the latter. The following are the results of these experiments:

"1. The transformation of potato-meal and of flour into sugar, by digestion in saliva at blood heat, was effected more rapidly in the concentrated than in the unconcentrated saliva. Transformation commenced in half an hour, and was completed in from two to three hours with an adequate supply of saliva

"2. The mass thus acted upon by concentrated saliva acquired equally with unconcentrated saliva the power of changing unboiled starch into sugar. Free acid is formed in a small quantity only, just as in the digestion of boiled starch in the stomach.

"3. Cheese digested with concentrated saliva is acted upon almost immediately, and changed in from two to three hours into a chyme-like mass.

"4. Cooked muscular fibre colliquesces in like manner, the fibrils melting away as I have figured in my previous work *De Aliment. Concoct.*, p. 7, and Fig. 1-8. The reduction, however, is not so rapid as that of cheese. This point appears to me of considerable importance, and is one not noticed in the accounts of the earlier attempts at artificial digestion. It must be observed that large portions of flesh are only chymified on the surface, so that the chyme thus formed must be removed by moving the portion under experiment, so that the subjacent parts may be duly exposed to the action of the digestive fluid. Minute pieces colliquesce much sooner than large. The concentrated saliva of the horse (which is very alkaline) digests flesh and cheese more readily, if the alkali it contains be previously neutralized either wholly or in part. On the other hand, the transformation of vegetable matters, as from starch to sugar, is more quickly effected by strong alkaline saliva.

"5. The saliva of the dog is less alkaline, and acts on animal fibre like the neutralized saliva of the horse.

"6. The digestion of animal fibre in the saliva of a horse is facilitated by mixing vegetable matters with it, as boiled starch." (p. 191-3.)

The rules for promoting digestion may be easily gathered from the preceding experiments. In the first place, the food must be well masticated. This rule every physician commends to his dyspeptic patients. But thorough mastication demands the exercise of good teeth and active jaws. The masseters rarely fail; the teeth often. The cause of dental decay is well hinted at by our author. Physiologically, the teeth are products of the epidermis, and their healthy condition is as much influenced by that of the mucous membrane of the alimentary canal, as the cuticle and hair are by the condition of the cutaneous organs. To brush and scrub the teeth is well enough when they are sound and the digestive powers are good; but when decay begins to take place attention must be directed to the digestive organs, if the progress of that decay is to be arrested. The first consideration will be not to slip the food down imperfectly masticated for fear of exciting toothach, but to comminute the food more minutely before putting it into the mouth, and then mixing it well with the saliva. It is exceedingly probable that the decayed teeth for which the Germans and Americans are nationally notorious, are dependent in a great degree on the too rapid ingestion of their food, so common with all classes of these people.

The secretion of saliva continues after completion of the meals, sometimes to an extraordinary amount. (De Alim. Concoct., p. 57.) To swallow the saliva thus secreted is the best, because natural, remedy against morbid acidity. If the post-prandial secretion be defective, sweetmeats are its best excitants.

In delicate persons the *temperature* of the stomach may be too low for proper chymification, and warm drinks should be taken. Too great a quantity also of acid may be formed towards the end of the process. If this be the case, soda-water is commendable, or the carbonate of soda, or "fluid magnesia," previously diluted, or a flow of saliva may be excited, and the product swallowed.

*Culture of chylification.* A few words under this head must suffice. The chyme passing into the duodenum is further transformed into the elements of chyle, namely albumen and fat. There the bile has a duty to perform with the chyme analogous to that of the saliva on the raw material. It de-oxydizes the oxydized constituents of the chyme;—this process failing, saccharine matter is taken up into the circulation from the bowels, as occurs in diabetes mellitus. Professor Schultz thus ascertained by actual experiments that one part of chyme requires two parts of bile to de-oxydate it; consequently, if the chyme passing into the duodenum after each digestion be estimated at six ounces, twelve ounces of bile must be excreted: now, as the gall-bladder can contain from one to two ounces only, the rest must come from the liver direct. So that the gall-bladder is only a receptacle for the bile secreted in the intervals of digestion.

*Moulting of the intestinal canal.* At the time we were amusing ourselves with Professor Schultz's queer title, Mr. Goodsir's observations on digestion and the absorption of chyle came under our notice, (see Br. and For. Med. Rev. vol. XIV, p. 566, and Dr. Carpenter's most interesting Report in last Volume). The curious fact stated by Mr.

Goodsir, namely, that there is a multitude of cells continually forming on the surface of the villi for a specific purpose, whose life is but of a very brief duration, and whose debris fall away into the chyme, seemed singularly in harmony with Professor Schultz's researches on the life and death of the blood-vesicles: and we must confess that we turned with no little eagerness to see whether in the section now under notice Professor Schultz took cognizance of these moulting vesicles of the villi, discovered by Mr. Goodsir. His general views are as follows:

"Hitherto the formation of fæces in the intestines has been looked upon as merely a mechanical separation of the indigestible portion of the food. Nevertheless, the vital process of chylification, and the moulting of the alimentary apparatus which commences in the mouth and salivary glands, and attains to its highest intensity at the lower end of the intestines, are closely connected with it. The alkaline salts of the saliva are neutralized by the acid in the stomach; the salivary matter appears for the most part to become ammonia, which also being neutralized in the stomach and large intestine, is carried downwards; and thus we can explain how it is that so large a quantity of acetate of ammonia and the nitrates are found in the excrements. The bile is precipitated by the acid of the chyme, and carried to the excrement as the so-called acid of the bile; and the intestinal mucus is no other thing than the moulting of the layer of the mucous membrane which becomes attached to the surface of the feces in the lower portion of the canal. This moulting is a necessary condition for the renewal of the intestinal mucous membrane, and its interruption is followed by cessation of all the functions; since the muscular layer becomes stretched, and the movements of the intestines interrupted. The secretion of a fat in the lower portion of the large intestines rich in carbon and smelling strongly is analogous to the biliary secretion. The uninterrupted progress of the intestinal moulting specially favours the process of renewal carried on during the formation of chyle, and its disturbance can cripple the whole vital process of assimilation. There are three things here to be specially considered in dietetics. 1. The reciprocal decomposition of the chyme and the bile. 2. The separation of the chyle from the products moulted during the process of its formation. 3. The purifying from mucus of the whole canal." (p. 200-1.)

According to Mr. Goodsir's observations great numbers of epithelium cells are being continually formed and moulted (in Schultzian phrase) during chylification. When the gut contains no chyme, the development of new vesicles ceases. During the interval of repose, the epithelium is renewed. We can only find room for one of the dietetic maxims Professor Schultz founds upon his researches: that one is this—If the stomach and cæcum be excited on the one hand into action, they must not on the other be deprived of their periodic refreshment and repose.

*Culture of the renewal of the blood. New experiments on the formation of blood.* This is an attractive title for all who may be half-past fifty; for the blood, as Professor Schultz observes, is really the fountain of life. How many sexagenarians sigh and audibly express their wish to have young blood in their veins!

We pass over the history of the youthifying process by transfusion of blood, to the more practical subject of the culture of the blood in its formation. This brings us back to the chyle and to digestion. The products of that process handed over to the lymphatic system for the renewal of the blood are albumen and fat. It has been ascertained by experiments of Majendie and others, that a variety of food is necessary to prolong life ; but the microscope had not been used for the purpose of ascertaining the changes induced in the organic constituents of the blood during these researches. Professor Schultz therefore made some experiments himself. Three dogs, fed with pure olive oil, all died within seven days : one on the 5th, one on the 6th, and the other was killed when in a dying state on the same day. On examining the latter, it was found that the oil had only been partly chymified. Numerous mucous cells were in the chyme found in the stomach. There was a small quantity of milk-white chyle in the lacteals and thoracic duct without any admixture, however, of the red vesicles. Examined under the microscope it was found to consist principally of distinct, perfectly round fat-globules. The plasma seemed imperfectly formed, and its coagulating power was weak. The plasma of the blood in the arteries was deeply reddened ; the vesicles slightly coloured, deprived of all turgescence, and collapsed in a remarkable manner, with angular, folded, and wrinkled forms, without granules, like dead vesicles many days old. There was a great number of oil-globules in the blood and bile, resembling those found in the lymph. It was evident also that oily deposits in the parenchyma of organs had taken place. The liver was impregnated with microscopic globules of oil, as was also the spleen. But the lungs were the most remarkably altered. They were collapsed, bloodless, and of a bright red colour. Oil-globules were deposited in the tissue connecting the bronchial ramifications with the blood-vessels in such quantities, that nothing but cells full of them were apparent, the bronchial cells being compressed by them : so that it is probable little of the oil was chymified, the remainder being absorbed by the veins, and mixed mechanically with the blood. Professor Schultz traces the whole series of phenomena described, and comes to the conclusion that no vesicles were formed round the lymph-globules. In consequence of this, the regular metamorphosis of the blood-constituents, and the development of the plasma were at once hindered.

Three dogs were fed with raw potato-flour. One of them died on the 10th day; the other two were killed on the 11th, when just dying. With the exception of the deposit of oil in organs, the changes observed, especially in the constituents of the blood, were as described above.

We have some details of experiments instituted to ascertain the amount of fat produced during chylification. The general result is that nearly one third of the chyle is transformed into fat.

*Formation of lymph-globules and vesicles.* The formation of lymph-globules is the first act of the vital process after the chyle has passed into the lymphatic vessels. A separation of the constituents of the lymph takes place into the thicker globules and the thin plasma of the lymph, the former having fat as their basis, the latter albumen. The formation of vesicles round the lymph-globules is the second act of the vital process. We have already alluded to this, and as Professor Schultz adds nothing of importance to his previous statements, we pass on to consider the next subject.

*Variations in the organic constituents of the blood in different classes of animals.* The blood-vesicles are most contractile in the carnivora, and are large but less numerous than in the herbivora. The quantity of colouring matter differs very much in different classes: in fact, this extreme variation has given origin to the division into white and red blooded. All animals have, however, red blood: it is the habitat of the colouring matter which makes the difference: *all invertebrata have white vesicles and coloured plasma*; *all vertebrata have coloured vesicles and white plasma.* This principle is uniformly applicable, for even the red-blooded invertebrates have white blood-vesicles.

*Ripening of the blood.* The ideas of Professor Schultz respecting the action of the lymphatic glands on the blood are curious. It will be obvious that the perfection of the blood depends on the respiratory powers of the blood-vesicles. The degree of organization in the organized constituents of the blood, is closely connected with the structure of the respiratory organs, and the mode in which the air is brought into contact with the circulating fluid. In the embryo the complete formation of the blood only commences with respiration. The great end of respiration is the organization of the plasma through the metamorphosis of the granules in the vesicles: at the same time the membranes of the vesicles attain their full growth and highest stage of development, as may be seen in the embryos of fishes, but more particularly of the amphibia: but on the other hand, if the organic constituents are imperfect the results of respiration are imperfect. In the water-breathing of fishes the development of the vesicles attains only the stage of lymph-development in the warm-blooded animals, particularly the mammalia. It is worthy notice, however, that animals breathing by gills have no lymphatic glands, and that the placental respiration of the fœtal state is precisely analogous to the permanent respiration through water, the blood carrying the oxygen in the one case, as the water in the other. Now, the lymphatic glands act after birth as placentæ, and so are subservient to the formation of lymph; but in all cases the development of the vesicles remains at an inferior stage, whether it be in the lymphatics in fœtuses, or in fishes. The embryos of birds present a remarkable exception, since the air is applied through the allantoid directly to the organic constituents, just as it is after birth.

The perfection of the blood may be prevented in various ways. If the plasma be too alkaline, or the contrary, its capacity for coagulation is diminished; it is too fluid, and the fatty matter of the lymph-globules and of the granules is not properly transformed. The plasma is, nevertheless, alkaline in health. Two drops of acetous acid neutralize one drachm of serum. The serum of a person labouring under diabetes required from a half to one minim only; of a scrofulous person only one drop to four drachms; and in another Professor Schultz found the serum quite neutral. In fact, the incapacity to coagulate was found to be in proportion to the want of alkalinity. The cause of this is the excessive formation of acid in the alimentary canal, which passes into the plasma, and neutralizes the alkali. The imperfect formation of granules depends upon an excess of carbonaceous food over the nitrogenized. Too much acid in the alimentary canal will have a similar effect by rendering the bile incapable of completing chylification, and so hindering the formation of fatty matter. Food in which nitrogen is deficient gives rise to imperfectly formed vesicles like those of the embryo. These absorb oxygen imperfectly, and remain light-coloured from the resulting deficiency in colouring matter. Such vesicles are also weak, and incontractile, and cannot give out the carbon they take up in the system; and for a similar reason they readily give out their colouring matter to the plasma, thus rendering the latter fluid an irritant to the capillaries, and giving rise to the so-called inflammatory diathesis. When with this want of contractility of the vesicles, the granules are rapidly and imperfectly developed, and the plasma remains albuminous and less fit for the formation of fibrine, we have the state which constitutes the hemorrhagic diathesis. In all those cases, the evolution of animal heat is less than natural. As a preponderance of vegetable food is followed by a too rapid development of the granules and debility of the vesicles, so a preponderance of animal food gives rise to a predominance of the vesicles over the granules; oxygen being absorbed more freely than is required, an inflammatory excitement is developed in the intestines, and in the capillaries of both the muscular and nervous systems. This condition is observable in gout and the gouty diathesis. Further, it is necessary that the organic constituents of the blood pass through their embryo state just as the embryo itself, before they can be perfectly developed: and as the lymphatic glands are the gills and placentæ of the system, if these perform their functions imperfectly, as in scrofulous constitutions, a deposit from the unripe blood takes place. Hence the development of chlorosis and phthisis.

Such are some of the general views of our author: for a more particular detail we must refer our readers to his book, promising them at the same time much amusement and some instruction from the perusal. Numerous practical rules in dietetics are laid down, as flowing from these principles. In referring to the culture of the blood, Professor Schultz states the curious general principle, that the blood-vesicles must be considered as true animal leaves. Like leaves, they have a period of birth, perfection, and decay, and just as the former may exist for a month, or a year, or many years, so may the blood-vesicles. The blood-vesicles of the amphibia have the slowest development and the longest life; those of birds, mammals and men, have the quickest development and shortest life. Under this head a new pathology of the blood is developed, and the causes of chlorotic, venous, melanotic, and bilious blood explained. Menstruation and hemorrhoidal flux are shown to be identical, and in close relation with, and complementary to the portal system. They carry off, in fact, the moulted effete vesicles, the sordes of the blood. Professor Schultz's views are supported by microscopic researches into the nature of these constitutional discharges. These we subjoin.

*Microscopic analysis of hemorrhoidal blood.* Only a small quantity, about a teaspoonful, could be collected. It was not reddened by oxygen or common salt and did not coagulate. The vesicles generally were filled with colouring matter, although a great portion of the latter was present also in the serous plasma. The dark red vesicles were for the most part without granules, were scarcely transparent even at the margins, which were collapsed, and fallen in, and did not contract on the addition of salt to the blood. There were a few freshly-formed vesicles here and there, but, altogether, the fluid strongly resembles portal blood.

*Analysis of the menstrual discharge.* A teaspoonful collected from a healthy female was neither a bright nor a dark red, but rather of a light dull red. The vesicles were heavy, and formed a precipitate which when the discharge was spread out on a plate formed to the naked eye a red cheese-like coagulum. Observed through the microscope this apparent coagulum was found to be composed of perfect vesicles heaped together, yet isolated, and not adherent to each other, as easily happens to the vesicles in living arterial and venous blood. Saline solutions reddened it a little, but almost imperceptibly. The vesicles swam about in a serous fluid not coagulable like the plasma, and somewhat reddened by colouring matter. They differed in colour, some were very small and colourless, others (the heaviest) were impregnated with colouring matter, and these were also the largest. The latter were also found to wrinkle when placed in a saline solution. In all, with the exception of some perfectly formed, unchanged vesicles, the granules are either altogether wanting or remarkably small. In the small, colourless, round vesicles, the membrane is drawn thickly round these little granules, so that they present the appearance of lymph-globules. The addition of iodine shows their real nature. From these and other less remarkable appearances, Professor Schultz is of opinion that the menstrual secretion is analogous to portal blood.

*Physiology and pathology of the water-cure.* Professor Schultz instituted experiments on horses and oxen, to ascertain the changes induced in the blood by water-drinking, and by mechanical dilution. We have not room for the details, but the general results are that estimating the mass of blood in a man at thirty pounds, 17·36 ounces of water may be added to the circulation by drinking it; and that in proportion as the blood becomes more watery a separation of the colouring matter from the vesicles is more extensive and its solution in the plasma more facile. In the water-cure three things are to be considered, namely, 1, the water itself, as water; 2, its temperature; and 3, its chemical constituents. Professor Schultz shows that the temperature is the main agent in the Priessnitz method, the alternation of cold and heat acting upon the skin, and inducing reaction and violent diaphoresis. In young constitutions and those in whom the organs are not structurally diseased, the diaphoretic method of Gräffenberg may be beneficial, but it cannot be considered otherwise than as a cruel and dangerous remedy for weak, elderly people. Perspirations induced by vigorous exercise, medicines being administered as auxiliary thereto, are from our experience exceedingly serviceable in those diseases in which the water-cure is found most beneficial, namely, chronic visceral disease in gouty subjects past middle age, or in individuals who have lived fast. When the separation of the colouring matter from the vesicles is indicated, means less irksome than the water-cure may be adopted. This indication is readily fulfilled, Professor Schultz asserts, by drinking acidulated drinks, in fact by the *acid*-cure. Melanotic, bilious cachexies have yielded to the ingestion of two quarts of acidulated lemonade daily: one quart to be taken in the forenoon, and another in the afternoon.

We shall now close this notice of Professor Schultz's work, with the observation that our limited space has not permitted us to do full justice to the new and very curious views it contains. We heartily commend it to the perusal of all who take an interest in the progress of scientific medicine. It is not free from many and great faults. Distant, very distant analogies are allowed to have the force of first principles : facts are mixed up in such a way with inferences that we have often found it impossible to separate the one from the other, and in the chapter on the renewal of the soul-life, downright assumptions and fanciful speculations are made the basis of grave psychical gymnastics and dietetics. Nevertheless, the book is a good book and a praiseworthy, and full of truths. Even if less interesting, it would be valuable as an antagonism to the Liebig school of bio-chemical philosophy.

---

